# Effect of topical *Zingiber cassumunar* on painful diabetic neuropathy: a double-blind randomized-controlled trial

**DOI:** 10.12688/f1000research.131344.2

**Published:** 2023-11-13

**Authors:** Nachapol Jatuten, Phuangthong Piyakunmala, Jiratha Budkaew, Bandit Chumworathayi

**Affiliations:** 1 Selaphum Hospital, Selaphum District, Roi Et, 45120, Thailand; 2 Social Medicine, Khon Kaen Hospital, Muang District, Khon Kaen, 40000, Thailand; 3 Obstetrics and Gynaecology,, Faculty of Medicine, Khon Kaen University, Khon Kaen, 40002, Thailand

**Keywords:** Painful diabetic neuropathy, Zingiber cassumunar, Plai balm

## Abstract

**Background:**

Plai or *Zingiber cassumunar* Roxb. was registered into the Thai Traditional Medicine list since 2011. However, there is limited evidence regarding Plai as a treatment in painful diabetic neuropathy (PDN). Therefore, this study aimed to evaluate the efficacy of topical *Zingiber cassumunar.*

**Methods:**

A RCT was conducted in patients with PDN during February to March 2019. All participants received oral gabapentin 300 mg before bed as a standard regimen. The intervention group (n=16) received Plai balm 15%w/w 0.5 gram to apply on their feet three times a day and the control group (n=15) received placebo balm to similarly apply. Pain score at baseline, 2 ^nd^ and 4 ^th^ weeks were assessed and compared. Patients’ quality of life, and adverse events, were collected. Mean pain scores before and after treatment in each group and between groups were also analyzed.

**Results:**

At the end of week two and week four, the Plai group showed statistically significant lesser mean pain scores than the placebo group by -1.47 (95%CI: -1.96 to -1.30, p-value < 0.001), and by -1.51 (95%CI: -1.92 to -0.13, p-value = 0.027), respectively. Moreover, the Plai group had more cases number/ percentages with at least 50% pain score reduction than the placebo group [12/16 (75%) vs 3/15 (20%), p-value = 0.004]. However, there was no statistically significant difference in quality of life between the two groups (overall p-value = 0.366). Adverse event was not found in any groups.

**Conclusions:**

*Zingiber cassumunar* balm (Plai) was efficacious for pain reduction in painful diabetic neuropathy.

**Registration:**

Registered with the Thai Clinical Trials Registry; TCTR20200221001.

## Introduction

Diabetic polyneuropathy is one of the complications of diabetes mellitus. There are many signs and symptoms, such as painful diabetic neuropathy (PDN), orthostatic hypotension, cardiac autonomic neuropathy, foot injuries, and wounds. ^1^ The incidence of diabetic patients who have developed neuropathy, is approximately 20%. In addition, 50-70% of them must undergo surgery for non-traumatic amputation. ^2^

PDN is the damage of nerves, that causes pain in the limbs. Patients will suffer from aching and feeling like shooting and stabbing pain. These symptoms will occur at the limbs during nighttime, and usually affect daily life activities, sleeping patterns and work, as a result, patient quality of life will be decreased. ^3^^,^^4^

Presently, non-steroidal analgesic medicines or opioids have been widely used for symptoms relieving. However, opioids are not only ineffective but have many side effects when treating PDN. Consequently, patients’ quality of life also decreases because of drug’s dosage-administration complexities, and long onset of action. ^5^^–^^8^ Capsaicin cream is effectively used for pain relief in PDN, but after a week, it might cause severe burn effect and finally require opioids for pain relief. ^9^

Plai or *Zingiber cassumunar* Roxb. is registered on the Thai Traditional Medicine list since 2011, ^10^ and categorized as a topical anti-inflammatory agent. The important extracted components from *Zingiber cassumunar* are (E)-4-(3, 4–dimethoxyphenyl) but-3-en-1-ol (compound D), (E)-1-(3, 4-dimethoxyphenyl) but-1, 3-diene (DMPBD) and zerumbone. ^11^ It has also been used for anti-inflammation, ^12^^–^^15^ pain relief, ^16^^–^^19^ and local anesthesia. ^20^^–^^23^

Although there were two successful studies of mixed multi-herbal preparation using *Zingiber cassumunar* Roxb. as its main component in treating diabetic foot ulcers, ^24^^,^^25^ there still has been no study regarding the use of Plai as a treatment in PDN. Therefore, this study aimed to evaluate the efficacy of a topical *Zingiber cassumunar* preparation by comparing with placebo on PDN.

## Methods

### Patients

This study was conducted at Selaphum Hospital, Selaphum District, Roi Et, Thailand. Participants were patients diagnosed with PDN. Neuropathic Pain Diagnostic Questionnaire (Thai version of DN4) was used for pain assessment, ^26^ and monofilament test was used as a sensory screening tool in these patients. ^8^ Written informed consent was obtained from all patients.

### Recruitment

Patients aged 20-60 years who had been diagnosed with diabetic mellitus for at least 1 year, tested for HbA1c within six months, suffering from pain intensity on a scale as moderate or more [Numerical Rating Scale (NRS), pain score ≥ 4], and with defined positive monofilament test result, were included. Patients who had been diagnosed with diabetic mellitus less than one year, with other caused neuropathy, clinically significant cardiovascular, foot ulcer and/or infection, pregnancy and lactation, and allergy to Plai, were excluded.

### Study design and oversight

This double-blind randomized-controlled trial was conducted within four weeks, from the 1 ^st^ of February to the 1 ^st^ of March 2019. All the authors were involved in the design and performance of the study, which was conducted according to the Declaration of Helsinki. The Khon Kaen Hospital Institute Review Board (KKHIRB) in Human Research (the oversighting IRB for Selaphum Hospital) approved the study protocol on 19 ^th^ December 2018 (KE61099). This trial was retrospectively registered with the Thai Clinical Trial Registry (TCTR20200221001) on 19 ^th^ February 2020, because “registration before recruitment” was missed, but authors finally registered it within one year after completion. Registration before recruitment is not a prerequisite for KKHIRB or Selaphum Hospital’s study. The study protocol did not differ from the registered one.

### Study treatment and procedures

Pain assessment was commenced at week 0 and indicated by using The World Health Organization Quality of Life (WHOQOL-BREF–THAI) ^27^ with NRS, pain characteristic, and pain mapping to follow-up side effect of Plai and its adverse events as baseline. Consequently, patients returned to clinic at the following week one, two, and four, to monitor and assess the accuracy of practice on frequency, and dosage of balms’ use. The patients’ quality of life in week 4 were observed and recorded.

Data about adverse events such as burning sensation and pruritus were collected by history taking and examination at week 1, 2, and 4, similar to the primary outcomes, ^8^^,^^26^ but edema/erythema/papules were collected by visual examination.

Block-of-four randomization was applied to this study, concealment was done by telephone calls to research assistants. Participants were divided into two groups. All participants received oral gabapentin of 300 mg, one tablet a day before bedtime as a standard regimen. The intervention group (n=16) received Plai balm 15% w/w 0.5 gram to apply on their feet three times a day and the control group (n=15) received the placebo balm to apply similarly.

### Preparation of the Plai balm and the placebo balm

To obtain the 100 grams of Plai balm, we mixed 15 grams of Plai oil with a balm base (which contained paraffin 10 g, Vaseline 50 g, camphor 10 g, Borneo camphor 5 g, menthol 5 g, and wintergreen oil 5 g). The placebo balm was made by increasing the first two components to paraffin 15 g and Vaseline 60 g, without adding Plai oil. Other active plants’ ingredients were still similar to the Plai balm. Both kinds of balm were loaded into a 60 g-sized case: 18 Plai balm and 17 placebo balm were labeled by numbers according to their randomization scheme (1-35). The contents inside the cases were not known to the test subjects or to the treating physicians.

### Endpoints

The mean differences of pain scores in NRS at week two and four between the two groups were the first endpoint. The other endpoint was the comparison of patients’ numbers/percentages who have at least 50% pain reduction at week two and four between the groups. Additionally, The World Health Organization Quality of Life (WHOQOL - BREF–THAI) scores changes at week four were compared between the groups.

### Statistical analysis

The preliminary study suggests that the number of participants should be 24-36 people. Consequently, this study recruited 35 participants, which were divided into two groups. Generalized estimating equation (GEE) was used to analyze the mean differences of pain scores at week two and four. Fisher’s exact test was used to analyze the differences in patients’ numbers/percentages who had at least 50% pain reduction at week two and four between the groups. Independent t-test was used to analyze the differences of The World Health Organization Quality of Life (WHOQOL-BREF–THAI) scores changes at week four between the groups. Mann-Whitney U-test was used to compare skew continuous data.

## Results

### Study patients

**Figure 1.**
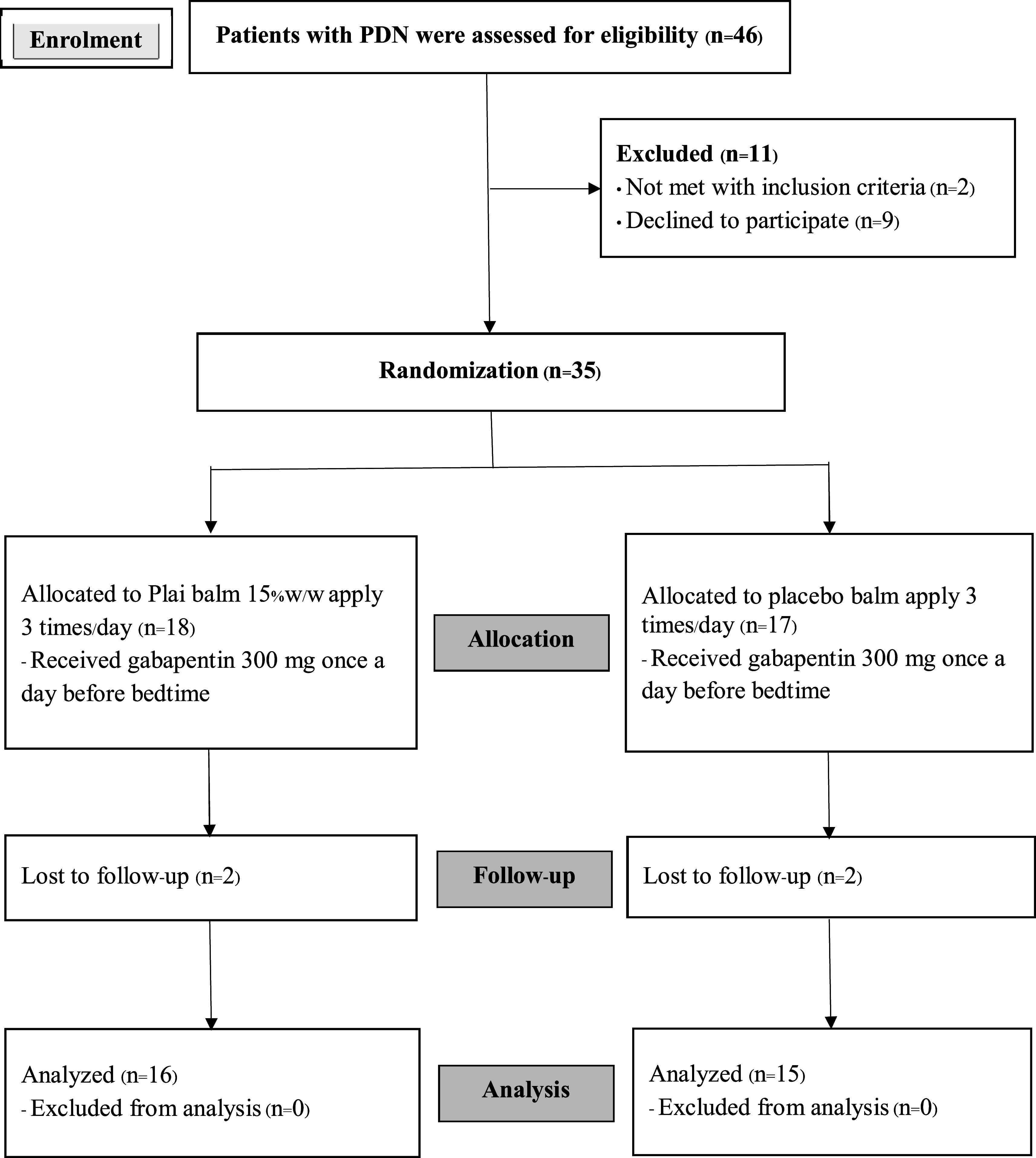
CONSORT flow diagram.

There were 826 diabetic mellitus type 2 patients treated at Selaphum Hospital during the study period. Although 88 of them had developed PDN, only 46 patients were able to participate according to the age criteria. Eleven of them were later excluded by other exclusion criteria (2 had foot ulcers, and 9 declined to participate). As a result, 35 of them were randomly assigned to each group. The intervention group had 18 participants receiving Plai balm, and the control group had 17 participants receiving placebo balm. Two participants in each group were later excluded due to follow-up loss ( Figure 1).

The patients’ characteristics, pain characteristics, and pain mapping of PDN at baseline, were similar among the two groups ( Tables 1, 1.1, and 1.2).

**Table 1.** Baseline characteristics of the patients.

Patients’ characteristics	Plai (n=16)		Placebo (n=15)		P-value
	Number	Percent	Number	Percent	
**Age (years)** ^**§**^					0.064
Lower than 50	2	12.50	4	26.67	
51 or more	14	87.50	11	73.33	
Mean (±SD)	55.56	(±5.43)	52.87	(±5.57)	
Median (min:max)	58	(39:59)	54	(39:59)	
**Gender** **					0.561
Male	7	43.75	6	40.00	
Female	9	56.25	9	60.00	
**Duration of diabetes (years)** ^**¶**^					0.611
Lower than 5	6	37.50	4	26.67	
6 or more	10	62.50	11	73.33	
Mean (±SD)	7.50	(±3.29)	8.07	(±2.81)	
Median (min:max)	6.50	(3:12)	8.00	(4:12)	
**Duration of PDN (years)** ^§^					0.662
Lower than 1	8	50.0	8	53.33	
2 or more	8	50.0	7	46.67	
Mean (±SD)	1.88	(±1.31)	1.67	(±1.05)	
Median (min:max)	1.5	(1:6)	8.00	(1:5)	
**History of hypertension** **					0.561
Yes	9	56.25	9	60.00	
No	7	43.75	6	40.00	
**HbA1c at screening** ^**¶**^					0.534
Mean (±SD)	11.09	(±2.14)	10.59	(±2.22)	
Median (min:max)	10.9	(8.3:16.6)	8.00	(7:15.1)	
**Baseline average daily pain** ^**§**^					0.280
Mean (±SD)	6.81	(±1.47)	6.33	(±1.59)	
Median (min:max)	6.5	(5:10)	6	(5:9)	
**NSAIDs**					0.525
Yes	2	12.50	1	6.67	
No	14	87.50	14	93.33	
**Opioids** **					0.475
Yes	1	6.25	2	13.33	
No	15	93.75	13	86.67	

PDN=painful diabetic neuropathy.

^¶^ Independent t-test.

^§^ Mann-Whitney U-test.

^**^ Fisher’s exact test.

**Table 1.1.** Baseline pain characteristics of PDN.

Pain characteristics	Plai (n=16)		Placebo (n=15)	
	Frequency	Intensity	Frequency	Intensity
	(Percent)	(Mean±SD)	(Percent)	(Mean±SD)
1. Throbbing	6 (37.5)	1.44±0.55	11 (73.33)	1.45±0.52
2. Shooting	12 (75)	1.92±0.79	12 (80.0)	1.33±0.49
3. Stabbing	10 (62.5)	1.70±0.67	4 (26.67)	1.75±0.5
4. Sharp	12 (75)	2±0.60	11 (73.33)	1.36±0.50
5. Cramping	4 (25)	2±0.81	5 (33.33)	2±0.71
6. Gnawing	6 (37.5)	1.83±0.98	6 (40.0)	1±0
7. Burning	7 (43.75)	1.86±0.38	11 (73.33)	1.45±0.68
8. Aching	7 (43.75)	1.43± 0.53	4 (26.67)	1.45±0.53
9. Heavy	5 (31.25)	1.6±0.55	4 (26.67)	2±0.81
10. Tender	3 (18.75)	1.67±0.58	7 (46.67)	2.75±0.5
11. Splitting	2 (12.50)	1.5±0.7	2 (13.33)	2.5±0.71
12. Exhausting	10 (62.5)	1.7±0.67	6 (40.0)	1.83±0.75
13. Fearful	6 (37.5)	1.83±0.75	3 (20.0)	2±1
14. Sickening	15 (93.75)	1.73±0.59	14 (93.33)	1.57±0.85
15. Punishing-Cruel	13 (81.25)	1.54±0.66	12 (80.0)	1.5±0.79

**Table 1.2.** Baseline pain mappings of PDN.

Pain mappings	Plai (n=16)		Placebo (n=15)	
	Number	Percent	Number	Percent
Posterior ankle pain	12	75.00	12	80.00
Lateral ankle pain	12	75.00	12	80.00
Medial ankle pain	10	62.50	10	66.67
Anterior ankle pain	10	62.50	10	66.67
Dorsal forefoot pain	16	100.00	15	100.00
Dorsal great toe pain	16	100.00	15	100.00
Dorsal lesser toe pain	16	100.00	15	100.00
Metatarsal head pain	13	81.25	15	100.00
Plantar great toe pain	16	100.00	15	100.00
Plantar lesser toe pain	12	75.00	14	93.33
Plantar midfoot pain	16	100.00	15	100.00
Heel pain	16	100.00	15	100.00

### Primary outcomes

The mean changes of pain scores in each group were significantly decreased. The mean changes of pain score in the Plai group decreased by -3.00 (95%CI: -3.58 to -2.41, p-value <0.001) at week two, and by -3.44 (95%CI: -3.87 to -3.00, p-value <0.001) at week four. The mean changes of pain score in the placebo group also decreased by -1.53 (-95%CI: 2.08 to -0.98, p-value <0.001) at week two, and by -1.93 (95%CI: -2.46 to -1.40, p-value <0.001) at week four ( Table 2).

**Table 2.** Mean changes of NRS scores in each groups.

Groups	Baseline Mean (SD)	Week 2 Mean (SD)	Week 4 Mean (SD)	Week 2		Week 4	
				Mean change * (95%CI)	P-value	Mean change * (95% CI)	P-value
**Plai**	6.81 (1.47)	3.81 (1.04)	3.38 (1.02)	-3.00 (-3.58 to -2.41)	<0.001	-3.44 (-3.87 to -3.00)	<0.001
**Placebo**	6.33 (1.59)	4.8 (1.57)	4.4 (1.40)	-1.53 (-2.08 to -0.98)	<0.001	-1.93 (-2.46 to -1.40)	<0.001

* Paired-T test.

Using GEE analyses by controlling confounding factors (age, sex, quality of life score), showed that the Plai group had statistically significant less pain at week two by -1.47 (95%CI: -1.96 to -1.30, p-value <0.001) when compared to the placebo group. At week four, the Plai group’s pain score was still decreasing and statistically significant less than the placebo group by -1.51 (95%CI: -1.92 to -0.13, p-value <0.027) ( Table 2.1).

**Table 2.1.** Mean of NRS scores differences between groups at week 2 and 4.

NRS differences	Week 2			Week 4		
	Plai (n=16)	Placebo (n=15)	P-value	Plai (n=16)	Placebo (n=15)	P-value
**Mean** ^**a**^ **(95%CI)**	-1.47 (-1.96 to -1.30)		0.001	-1.51 (-1.92 to -0.13)		0.027

^a^ Generalized estimating equations (GEE).

### Secondary outcomes

Numbers/percentages of patients who had at least 50% pain reduction of pain by NRS scores at week four were found significantly more in the Plai group when compared to the placebo group [12/16 (75%) vs 3/15 (20%), p-value = 0.004] ( Table 3).

**Table 3.** Numbers/percentages of patients who had at least 50% pain reduction in each groups at week 4.

Pain reduction amount	Groups		P-value
	Plai, n (%)	Placebo, n (%)	
**≥50%**	12 (75.00)	3 (20.00)	0.004
< **50%**	4 (25.00)	12 (80.00)	

The overall integration of WHOQOL- BREF–THAI 4 components was used. Physical domain, psychological domain, social relationships, and environmental domain analyzed patients’ quality of life scores at week four. Overall and each domain’s patients’ quality of life scores were no different between the two groups ( Table 4).

**Table 4.** WHOQOL-BREF–THAI scores in each groups at week 4.

WHOQOL-BREF domain	$\bar{X}$	SD	Mean difference	95%CI	P-value
**Physical**					
Plai	14.12	0.60	-1.74	-3.49 to 0.01	0.0504
Placebo	15.87	0.60			
**Psychological**					
Plai	20.56	0.96	-0.30	-3.21 to 2.60	0.832
Placebo	20.86	1.05			
**Social**					
Plai	10.875	0.24	-0.32	-0.99 to 0.34	0.329
Placebo	11.2	0.22			
**Environmental**					
Plai	26.25	0.61	0.52	-1.25 to 2.28	0.555
Placebo	25.73	0.61			
**Overall**					
Plai	71.81	1.48	-1.85	-5.98 to 2.28	0.366
Placebo	73.67	1.37			

### Adverse events

No adverse events such as edema, erythema, papules, pruritus, and burning sensation, were found in this study.

## Discussion

This double-blind randomized-controlled trial was conducted in PDN patients who had pain by NRS scores of four or more within four weeks, from the 1 ^st^ February to the 1 ^st^ March 2019 at Selaphum Hospital, Selaphum District, Roi Et, Thailand. Majority of participants were elderly female patients who had been diagnosed with poor controlled type 2 diabetic mellitus for years. Those patients met the criteria of having PDN, which obviously presented similar pain characteristics and pain mapping between the groups.

Based on this study’s method, all participants received oral gabapentin 300 mg, 1 tablet daily before bed as a standard regimen. The use of Plai balm 15% w/w 0.5 gram significantly showed pain reduction on PDN. The pain scores by NRS were decreased more in the Plai group compared to the placebo group [by mean differences of -1.47 (95%CI: -1.96 to -1.30, p-value <0.001) at week two, and by mean difference of -1.51 (95%CI: -1.92 to -0.13, p-value = 0.027) at week four]. These results derived from the effects of Plai balm on PDN.

Until now (December 2022), there has been no other trial that studied the efficacy of Plai on reducing pain in PDN. However, previous studies had shown that Plai being main component in mixed herbal preparations, is safe ^24^ and effective ^25^ in treating diabetic foot ulcers. Moreover, Plai has local anesthetic ^20^^–^^23^ and neuroprotective ^28^ effects, in addition to its effects on anti-inflammation ^12^^–^^15^ and pain relief. ^16^^–^^19^ These mechanisms in combination, might be the cause of pain reduction. Nevertheless, this study has been the first one to show the effect on PDN. Whether a neuroprotective effect also occurs in the peripheral nerves, is needed to be investigated.

In this study, not only Plai was found to be effective in pain reduction, but also safe with no adverse event reported, neither redness, swelling, burning or rash. These were similar to a systematic review done in 2017 by Chongmelaxme *et al.* ^15^ Even in two more recent studies, with very similar application but more mixed herbs, adverse events were still not found. ^24^^,^^25^ However, quality of life scores were not significantly different between groups at week 4, even though the quality-of-life scores were improved in each groups. This could be explained by the standard treatment both groups had received: 300 mg gabapentin, once a day. This might be also the main reason for similar changes in the quality-of-life scores.

Strengths of this study were 1) This has been the first RCT comparing treatment efficacy of Plai to placebo, 2) Pain reduction was found significantly different between the groups, and 3) Pain and quality of life scores were measured, reported, and analyzed using their standard methods. Weaknesses of this study might be that; 1) It has low sample size, 2) Some patients were lost from each group, and 3) Cost-effectiveness was not collected and analyzed. Future research with similar objectives may be conducted with larger sample sizes and more diverse settings.

Limitations of this study included 1) Authors could not know the reasons of losing following-up in four patients, and this might be from serious adverse events, and 2) Authors did not added any color or smell to the placebo balm to make it more similar to the Plai balm like Klaphajone *et al.* ^18^ However, both of them were only slightly different in their color and smell, and the color or smell added may cause allergy. If the treating physicians and patients did not open the case to campare them directly, it was very hard to differentiate.

## Conclusion

In conclusion, *Zingiber cassumunar* balm (Plai) was efficacious and safe for reducing pain in patients with painful diabetic neuropathy (PDN). However, quality of life scores changes in the Plai group were not significantly different from the placebo group.

## Data Availability

Figshare. Zingiber cassumunar Dataset. DOI: https://doi.org/10.6084/m9.figshare.21805182.v1. ^29^ This project contains the following data:
- This is the dataset of the research titled “Effect of topical *Zingiber cassumunar* on painful diabetic neuropathy: A double-blind randomized-controlled trial” Figshare. Zingiber cassumunar Protocol. DOI: https://doi.org/10.6084/m9.figshare.22180675.v1. ^30^
- This is the protocol of an RCT on Zingiber cassumunar for painful diabetic neuropathy (PDN). Data are available under the terms of the Creative Commons Attribution 4.0 International license (CC-BY 4.0). Figshare. Zingiber CONSORT Checklist. DOI: https://doi.org/10.6084/m9.figshare.22046570.v1. ^31^ Data are available under the terms of the Creative Commons Attribution 4.0 International license (CC-BY 4.0).
