# Supplementary material for: Effect of topical Zingiber cassumunar on painful diabetic neuropathy: a double-blind randomized-controlled trial
Source: F1000Res. 2023 Nov 13;12:334. Originally published 2023 Mar 24. [Version 2] doi: 10.12688/f1000research.131344.2 (PMC11364968; doi:10.12688/f1000research.131344.2)
Supplement: Peer review history

# Reviewer report: Reviewer response for version 2

**DOI:** 10.5256/f1000research.158673.r312657

**Published:** 2024-08-30

**Authors:** Arif Nur Muhammad Ansori

**Affiliations:** 1 Universitas Airlangga, Surabaya, East Java, Indonesia

**Relevance to Traditional Medicine**: The study focuses on *Zingiber cassumunar* (Plai), which has been recognized in the Thai Traditional Medicine list since 2011. This adds significant relevance to the research, as it explores a plant with established traditional use.

**Scientific Validation**: Evaluating the efficacy of topical *Zingiber cassumunar* is crucial for bridging the gap between traditional knowledge and modern medicine. The study contributes to validating and potentially integrating traditional remedies into contemporary therapeutic practices.

**Potential Impact**: If the study demonstrates the efficacy of *Zingiber cassumunar*, it could lead to broader acceptance and usage of this traditional remedy, benefiting both healthcare providers and patients seeking alternative or complementary treatments.

Is the work clearly and accurately presented and does it cite the current literature?

Yes

If applicable, is the statistical analysis and its interpretation appropriate?

Yes

Are all the source data underlying the results available to ensure full reproducibility?

Yes

Is the study design appropriate and is the work technically sound?

Yes

Are the conclusions drawn adequately supported by the results?

Yes

Are sufficient details of methods and analysis provided to allow replication by others?

Yes

Reviewer Expertise:

Plant Extracts

I confirm that I have read this submission and believe that I have an appropriate level of expertise to confirm that it is of an acceptable scientific standard.

# Reviewer report: Reviewer response for version 2

**DOI:** 10.5256/f1000research.158673.r222718

**Published:** 2023-11-16

**Authors:** Fifteen Aprila Fajrin

**Affiliations:** 1 Clinical and Community Department, Faculty of Pharmacy, Universitas Jember, Jember, East Java, Indonesia

Thank you for the revision, I think it is detailed enough to answer all my questions.

Is the work clearly and accurately presented and does it cite the current literature?

Partly

If applicable, is the statistical analysis and its interpretation appropriate?

Yes

Are all the source data underlying the results available to ensure full reproducibility?

Yes

Is the study design appropriate and is the work technically sound?

Yes

Are the conclusions drawn adequately supported by the results?

Yes

Are sufficient details of methods and analysis provided to allow replication by others?

Yes

Reviewer Expertise:

Molecular Pharmacology, neuropharmacology.

I confirm that I have read this submission and believe that I have an appropriate level of expertise to confirm that it is of an acceptable scientific standard.

# Reviewer report: Reviewer response for version 1

**DOI:** 10.5256/f1000research.144178.r193436

**Published:** 2023-08-21

**Authors:** Fifteen Aprila Fajrin

**Affiliations:** 1 Clinical and Community Department, Faculty of Pharmacy, Universitas Jember, Jember, East Java, Indonesia

I think the author has conducted this research well. But it needs some explanation for the following area:

1. There is no detail about the preparation of topical Zingiber cassumunar. And what is the composition of Plai balm, just Zingiber cassumunar or there are some active plants?
2. The author needs to explain why patients were excluded from this research and give detailed explanations.
3. In the result, I found the adverse events, but in the methods, there was no description of how this test was performed and also the assessment.
4. Almost half of the references are too old, the author can choose another reference that is more up-to-date.
5. The author needs to explain the limitation of this study.

Is the work clearly and accurately presented and does it cite the current literature?

Partly

If applicable, is the statistical analysis and its interpretation appropriate?

Yes

Are all the source data underlying the results available to ensure full reproducibility?

Yes

Is the study design appropriate and is the work technically sound?

Yes

Are the conclusions drawn adequately supported by the results?

Yes

Are sufficient details of methods and analysis provided to allow replication by others?

Yes

Reviewer Expertise:

Molecular Pharmacology, neuropharmacology.

I confirm that I have read this submission and believe that I have an appropriate level of expertise to confirm that it is of an acceptable scientific standard, however I have significant reservations, as outlined above.

# Response

**Published:** 2023-11-07

**Authors:** Bandit Chumworathayi

**Affiliations:** Khon Kaen University, Thailand

Responses to Reviewer #1

 Thank you very much for your kind comments and suggestions. Authors would like to response them as follows.

 1.0. I think the author has conducted this research well. But it needs some explanation for the following area:

 Response: Thank you for your admiration that we did this research well. More explanations are followed.

 1.1. There is no detail about the preparation of topical Zingiber cassumunar. And what is the composition of Plai balm, just Zingiber cassumunar or there are some active plants?

 Response: The detail of preparation was added to the Methods, and the composition of Plai balm was as the following table, other active plants (paraffin, Vaseline, camphor, Borneo camphor, menthol, and wintergreen oil) were similar, but with more paraffin and Vaseline added, to the placebo.

**Composition**

**Plai balm 15%w/w**

 Paraffin 10 g

 Vaseline 50 g

 Camphor 10 g

 Borneo camphor 5 g

 Menthol 5 g

 Wintergreen oil 5 g

 Plai oil 15 g

**Placebo**

 Paraffin 15 g

 Vaseline 60 g

 Camphor 10 g

 Borneo camphor 5 g

 Menthol 5 g

 Wintergreen oil 5 g

 Plai oil 0 g

 1.2. The author needs to explain why patients were excluded from this research and give detailed explanations.

 Response: From 11 excluded patients, the detailed explanations, that were lost, are as follows, and we have added them to the Results.

 • Did not meet inclusion criteria (foot ulcer, n=2)

 • Declined to participate (n=9)

 1.3. In the result, I found the adverse events, but in the Methods, there was no description of how this test was performed and also the assessment.

 Response: Data about adverse events such as burning sensation and pruritus were collected by history taking and examination at week 1, 2, and 4, similar to the primary outcomes [references number 8 (monofilament test) and 26 (Thai version of DN4)], but edema/ erythema/ papules were collected by visual examination when patient followed up. We have added these processes into the Methods (Study treatment and procedures).

 1.4. Almost half of the references are too old, the author can choose another reference that is more up-to-date.

 Response: On November 7, 2023, we have re-searched for relevant published papers in PubMed by using the terms “Zingiber cassumunar” AND “painful diabetic neuropathy”, and retrieved zero results. We re-tried with only “Zingiber cassumunar”, and retrieved 85 but no one was relevant. We have tried again in “Google Scholar”, but no one was relevant in this topic too.

 1.5. The author needs to explain the limitation of this study.

 Response: In addition to weaknesses, we have added accordingly in the last paragraph of the Discussion.
